# Glaucoma-TrEl: A web-based interactive database to build evidence-based hypotheses on the role of trace elements in glaucoma

**DOI:** 10.1186/s13104-022-06210-0

**Published:** 2022-11-18

**Authors:** Jyoti Kant Choudhari, Martin Eberhardt, Tanushree Chatterjee, Bettina Hohberger, Julio Vera

**Affiliations:** 1 Raipur Institute of Technology Raipur, Chhattisgarh, India; 2 Laboratory of Systems Tumor Immunology, Dept. of Dermatology, Universitätsklinikum Erlangen and Friedrich-Alexander-Universität Erlangen-Nürnberg, Erlangen, Germany; 3 Augenklinik, Universitätsklinikum Erlangen and Friedrich-Alexander-Universität Erlangen- Nürnberg, Erlangen, Germany

**Keywords:** Oxidative stress, glaucoma, Optic nerve, Intraocular pressure, Protein-trace element interactions

## Abstract

**Objective:**

Glaucoma is a chronic neurological disease that is associated with high intraocular pressure (IOP), causes gradual damage to retinal ganglion cells, and often culminates in vision loss. Recent research suggests that glaucoma is a complex multifactorial disease in which multiple interlinked genes and pathways play a role during onset and development. Also, differential availability of trace elements seems to play a role in glaucoma pathophysiology, although their mechanism of action is unknown. The aim of this work is to disseminate a web-based repository on interactions between trace elements and protein-coding genes linked to glaucoma pathophysiology.

**Results:**

In this study, we present Glaucoma-TrEl, a web database containing information about interactions between trace elements and protein-coding genes that are linked to glaucoma. In the database, we include interactions between 437 unique genes and eight trace elements. Our analysis found a large number of interactions between trace elements and protein-coding genes mutated or linked to the pathophysiology of glaucoma. We associated genes interacting with multiple trace elements to pathways known to play a role in glaucoma. The web-based platform provides an easy-to-use and interactive tool, which serves as an information hub facilitating future research work on trace elements in glaucoma.

## Introduction

Glaucoma is an optical neuropathy affecting the interpupillary and parapupillary area of the optic nerve’s (ON) head and the retinal nerve fiber layer [1]. The most validated risk factor for glaucoma is the rise of IOP [2]. The literature contains abundant data suggesting the multifactorial nature of glaucoma, including its connection to vascular changes [3, 4], interrupted retrograde transport of neurotrophins [5], ocular ischemia [6], and oxidative stress [7]. Several lines of evidence suggested that oxidative stress harm the trabecular meshwork (TM), resulting in an increased IOP and injury of the retinal ganglion cells (RGC), which provokes cell death and ON damage [8]. Oxidative stress refers to the damage caused by the over-activity of oxidising agents to cellular components [9]. It is caused by an imbalance between production and mitigation of reactive oxygen species, and it depends on exogenous/endogenous factors and the ability of cells to detoxify these substances [10, 11]. Surface abnormality ratio is a significant macular factor in the first phase of glaucoma pathogenesis for high-tension glaucoma, in which free radicals affect the TM endothelial cells and cause morphologic and functional changes inflicting diffuse injury [12]. Trace metal concentrations in the body could alter affect oxidative stress balance in the pathogenesis of glaucoma [13]. A higher blood ferritin level was linked to a higher risk of glaucoma in a South Korean cohort. Since serum ferritin, an iron containing protein, is connected to oxidative stress and inflammation, it might have a role in glaucoma development [14, 15]. In one clinical study, the authors measured levels of Cd, Fe, Mn, Co, Cu, and Zn in aqueous humor from patients with primary open angle glaucoma, pseudoexfoliation glaucoma, or cataract as controls. They identified changes in Zn or Fe concentrations as a putative cause or effect of glaucoma [16]. Additionally, increased levels of Zn can inhibit the absorption of Cu and cause Fe and Cu imbalances, which are critical to neuronal growth and immunity. Also, the increase of Zn levels can be a compensating mechanism for the neutralisation of free radicals which show elevated concentrations in glaucoma [17, 18].

Trace elements are linked to deregulation of certain glaucoma coding genes and their products. The levels of certain iron and/or copper-related proteins increased in glaucoma [19]. Yefimova et al. conducted a study to look at the distribution of iron and ferritin in the adult rat retina. They quantified iron concentration in the choroid and retinal pigmented epithelial cell layer, as well as in the inner segments of the photoreceptors and the outer retina and found that iron and ferritin shared similar distribution patterns in these tissues. Also, they found that the transferrin receptor likely helps transfer iron to photoreceptors [20]. The Ceruloplasmin (Cp) protein showed increased expression in the retina, RGC and inner nuclear layers after optic nerve crush in humans [21] [22] and mice [23]. Cp is involved in copper transport and is mainly synthesized in the liver [24]. The HFE H63D gene alteration has been documented to be linked to retinopathy in diabetes patients [25]. Intestinal iron absorption is significantly increased due to a mutation in HFE gene, and iron overloaded states can have significant implications in vision [26]. The cellular uptake of iron and cadmium utilizes similar transport pathways. Thus, gene alterations affecting iron-processing proteins (e.g. HFE H63D) may affect as well cadmium metabolism [27]. Some studies found alterations in the expression of iron-regulating genes in monkey and human glaucoma [19]. CBP is a ubiquitously expressed protein cofactor of the transcription factor CREB and regulates the development of eyes in drosophila [28]. Interestingly, mutations in the PHD type zinc finger domain of CBP reduce its transcriptional activity [29] [30]. Also, imbalances in zinc metabolism can alter the activity of zinc finger proteins [54].

Taken together, recent publications suggest that trace elements play an important role in the regulation and activity of proteins linked to glaucoma pathophysiology. In this context, we comprehensively investigate interactions between glaucoma-related proteins and trace elements and present the results in a web-based database.

## Implementation

We designed a workflow for the systematic detection of interactions between trace elements and protein-coding genes that display differential expression in glaucoma. To this end, we retrieved the trace element-binding proteins’ interactions from databases like MINAS [31], MetalPDB [32], RCSB [33], and MetalMine [34]. To integrate the information contained in the databases, we formulated a score, in which. a given trace element a protein-coding gene gets a score which is the number of databases in which their interaction is contained. Next, we filtered out genes with a score below two. The selected genes were annotated with relevant information including gene symbol, gene name, location and synonyms taken from Ensembl [35], protein ID, protein name and synonyms taken from RCSB [33] and UniProt [36], and interactions taken from MMDB [37]. We linked the GeneCards database [38] as well as the Protein Data bank in Europe (PDBe) [39] to integrate more information. Further and whenever possible, interactions between protein and trace element were annotated with the PubMed identifier (PMID) of the linked publication. We utilised ClueGO to annotate the genes with gene ontologies (GO) and link them individually to biological processes and pathways [40].

**Fig. 1:**
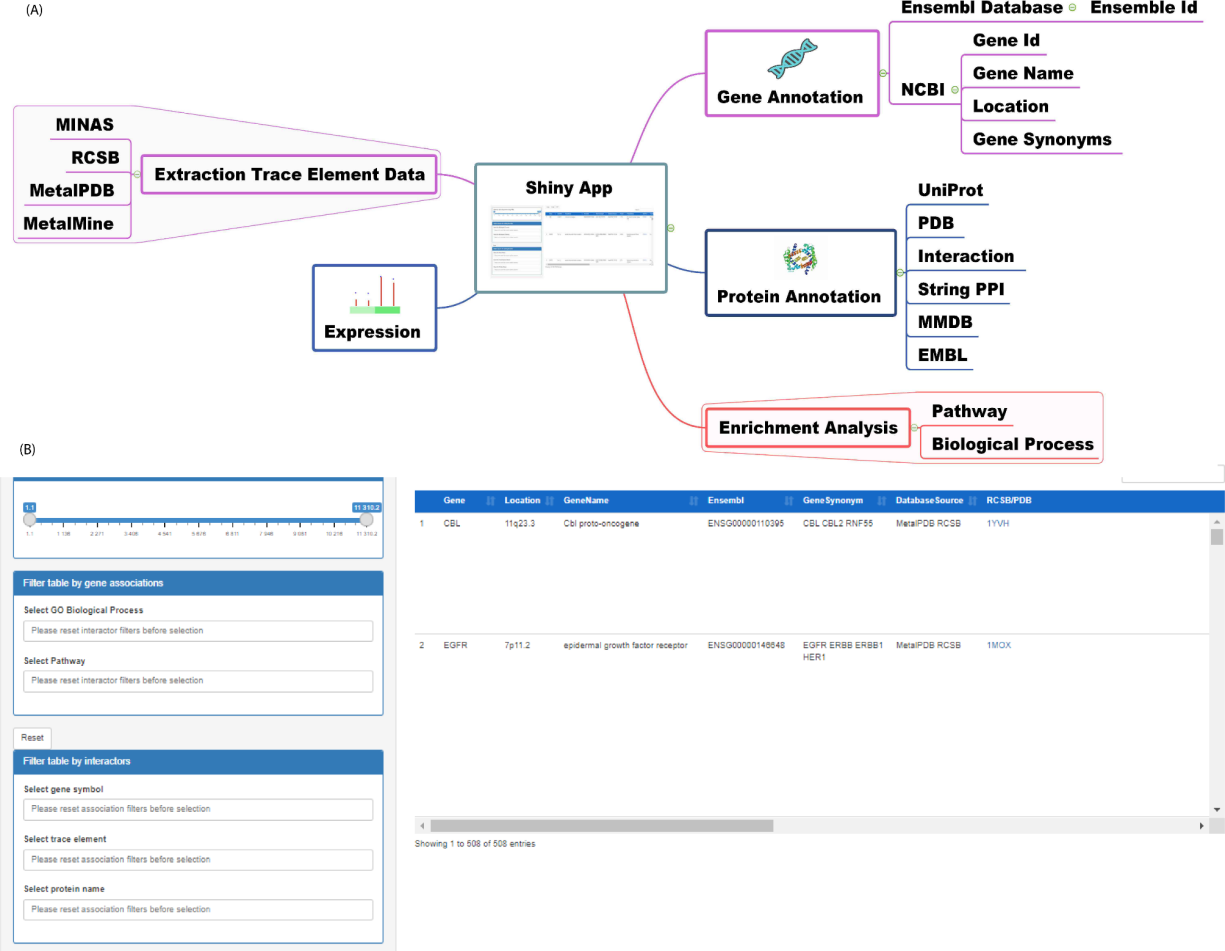
(**A**) Sketch of the workflow to construct Glaucoma-TrEl. (**B**) Screenshot of the Shiny R application. On the left, there is a slider for selecting genes based on expression in TPM. Further, one can select interactions based on (**1**) a gene or protein list (when empty, all genes are displayed), (**2**) a trace element (filter the genes based on whether they have known interactions with the selected trace element), or (**3**) the attribution of the genes to selected pathways or biological processes. The data are displayed in the table on the right-hand side

To investigate the expression of the protein-coding genes in glaucoma, we obtained and processed published RNA-Seq datasets of optic nerve head of feline congenital glaucoma (FCG) cats and age-matched disease-free cats (GSE110019) [41]. We selected genes showing an expression level equal to or greater than 1 transcript per million (TPM) in at least one of the conditions compared. Genes without interactions with trace elements were excluded from the table. We employed ClueGO to calculate a list of significantly over-represented GO terms related to the genes belonging to the database and obtain GO terms according to their similarity in the ontology [40]. All the information was incorporated in the database, which was implemented as a Shiny R app (Fig. 1).

**Fig. 2:**
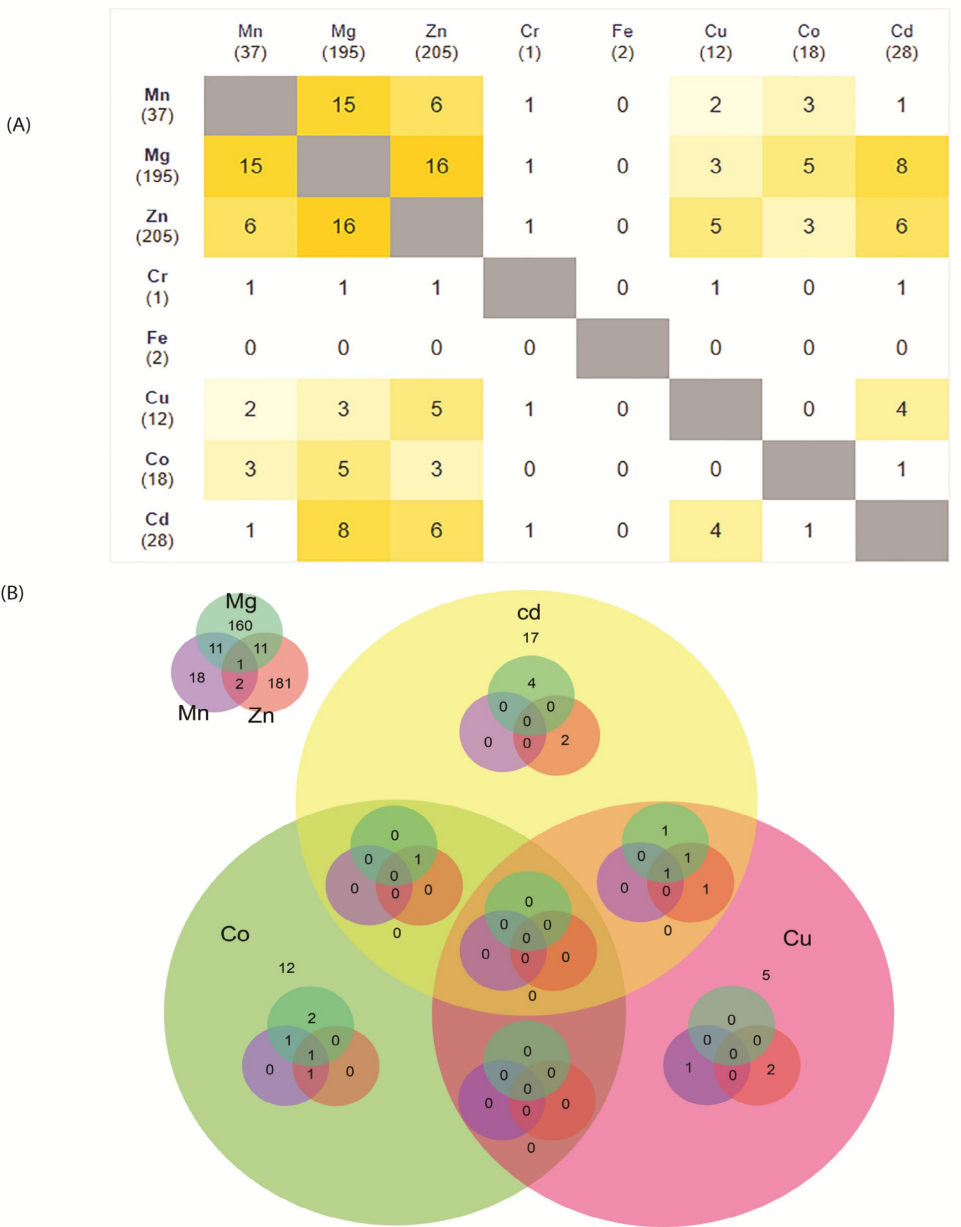
**A**) Pairwise trace element intersections accounting for mutual shared interactions with glaucoma-related genes. The matrix is symmetrical regarding the gray diagonal. A yellow color gradient is employed to indicate trace elements with higher number of shared interactions with coding genes. Also, columns and rows indicate in brackets the total number of interactions for each trace element. **B**) Venn diagram depicting the number of shared genes between six distinct trace elements. Chromium and iron are not shown due to their low number of interactions with the selected genes

## Results

We built a web-based interactive database containing information about interactions between trace elements and proteins in the context of glaucoma. To this end, we systematically collected and merged information existing in multiple databases, and integrated this with published expression data from glaucoma models. The web-based tool is based on Shiny [42], a computational framework that makes it possible to develop interactive web applications using scripts implemented in the R language (R Core Developer Team, 2013).

In the web interface, the full table can be browsed and downloaded, or it can be filtered by selecting a gene, trace element, or biological process/pathway. The search generates a table that aggregates and visualises multiple types of information. Precisely:

(i) Gene-related information like gene name, Human Gene Name Consortium symbol, and identifiers in databases like Ensembl, but also the list of pathways and biological processes linked to the gene according to GO Terms. Furthermore, the browser visualises the average TPM in the dataset utilised for filtering protein-trace element interactions (see Sup. Mat.).
(ii) Protein information like protein name, identifiers in relevant databases like PDB, EBML and Uniprot, and a STRING subnetwork of first neighbor protein-protein interactions [43].
(iii) Information describing the protein-trace element interaction like the databases in which the interaction is listed, the PMID for linked publications, and additional information like details about the binding site.
(iv) Other information like enriched pathway and biological process GO terms, which are associated to the gene interacting to a given trace element.

The online database consists of one table filterable by trace element, protein, pathway and BP terms. Figure 1B captures the user-visible front end of the app. Our database covers eight trace elements, namely Cr, Cd, Mn, Mg, Fe, Co, Cu, and Zn. The complete list holds 437 unique genes expressed in the analysed datasets and linked to eight trace elements. We also observed that 44 genes contain at least two trace element overlaps (Fig. 2B; Table 1). Eight genes had three or four interactions (APP, HDAC8, UBC, B2M, PDE4B, POLH, SNRPA, and SOD1), and the POLB gene interacts with six different trace elements. The most promiscuous trace element is Zn (205 interactions with glaucoma-related genes), followed by Mg (195), Mn (37) and Cd (28). For the identified trace element-gene interactions, in Fig. 2 A, we computed a pairwise intersections matrix comparing how many of their interaction partners are shared by any pair of trace elements. Sixteen genes interact with both Zn and Mg, while fifteen genes have interactions with Mg and Mn (Fig. 2). The Venn diagram in Fig. 2B shows the intersection of interacting genes between the trace elements, with Mg, Mn and Zn sharing a relevant number of mutual interactions. Interestingly, pathway enrichment analysis revealed that the 44 genes in Table 1 are associated with L1 signal transduction, chromatin modifying enzymes, DDX58/IFIH1-mediated activation of interferon-alpha/beta, and MET pathway negative regulation.

**Table 1:** Comparative analysis of multiple trace element-interacting genes

S. N.	Gene	#Int.	Interactors	S. N.	Gene	#Int.	Interactors
1	POLB	6	Mn, Mg, Zn, Cr, Cu, Cd	23	KDM6B	2	Mg, Co
2	APP	4	Mg, Zn, Cu, Cd	24	KRAS	2	Mg, Cd
3	HDAC8	4	Mn, Mg, Zn, Co	25	PABPC1	2	Zn, Cd
4	UBC	4	Mg, Zn, Co, Cd	26	PDE5A	2	Mg, Zn
5	B2M	3	Mg, Cu, Cd	27	PLK1	2	Mg, Zn
6	PDE4B	3	Mn, Mg, Zn	28	POLI	2	Mn, Mg
7	POLH	3	Mn, Zn, Co	29	POLL	2	Mn, Mg
8	SNRPA	3	Mn, Mg, Co	30	POLM	2	Mn, Mg
9	SOD1	3	Zn, Cu, Cd	31	POLR2A	2	Mg, Zn
10	ADAMTS1	2	Mg, Cd	32	PPIP5K2	2	Mg, Cd
11	ALDH2	2	Mn, Mg	33	PRIM1	2	Mn, Mg
12	BPHL	2	Mn, Mg	34	PRKCI	2	Mn, Mg
13	CD59	2	Zn, Cu	35	RAB5A	2	Mg, Co
14	DPP3	2	Mg, Zn	36	RAN	2	Mg, Zn
15	DUT	2	Mg, Zn	37	RPS27A	2	Zn, Cd
16	EGFR	2	Mg, Cd	38	SAMHD1	2	Mn, Mg
17	EPRS	2	Mg, Zn	39	SMYD3	2	Mg, Zn
18	FDFT1	2	Mn, Mg	40	SYT1	2	Mn, Cu
19	FDPS	2	Mn, Mg	41	UBB	2	Mg, Zn
20	FTH1	2	Zn, Cu	42	UHRF1	2	Mg, Zn
21	FTO	2	Mn, Zn	43	WRN	2	Mn, Mg
22	HAGH	2	Mn, Zn	44	ZCCHC6	2	Mg, Zn

## Discussion and conclusions

In developing countries, glaucoma causes irreversible visual impairment leading to vision loss in aged populations [51]. Glaucoma has been traditionally associated to hereditary genetic predisposition and increase of the IOP, but recent discoveries suggest that glaucoma is a multifactorial disease whose onset is controlled by a network of interconnected proteins and pathways. Further, imbalance in the bioavailability and metabolism of some trace elements has been associated to glaucoma [52]. Trace elements are integrated or regulate the activation of many proteins like in case of proteins containing zinc finger domains. Also, trace elements are implicated in protein modifications mediated by oxidative stress, and thus involved in the pathogenesis of glaucoma [15]. Thus, to systematically record the interactions of trace elements with glaucoma-associated genes can help elucidating the pathogenesis of this disease.

The eight selected trace elements have been reported in the serum and aqueous humor of glaucoma patients [53]. We found interactions between the eight trace elements and 437 unique genes. Interestingly, 44 genes interact with least two trace elements. Further, the *POLB* gene interacts with six trace elements and is linked to DNA damage repair. Upregulation of this gene in the retinal ganglion cell layer has been suggested as an early change in nerve injury rat models for glaucoma [44]. Eight more genes have three or four interactions with trace elements (*APP*, *HDAC8*, *UBC*, *B2M*, *PDE4B*, *POLH*, *SNRPA* and *SOD1*). Some members of the histone deacetylase family of proteins, to which *HDAC8* belongs, have been linked to retinal ganglion cell death after acute optic nerve injury [45]. *UBC* is a ubiquitin related to protein degradation and DNA repair, which has been identified in gene association studies in primary open angle glaucoma [46]. *B2M* is a component of MHC-I with a soluble variant linked to interferon gamma and RET signaling and present in the tear proteome, which has been used as marker for patient stratification in glaucoma clinical trials [47]. RET is a member of the receptor tyrosine kinase superfamily, which acts as receptor for members of the glia cell-derived neurotrophic factor family of ligands like GDNF and Neurturin. These ligands have an important role in the survival and differentiation of neurons and neoplastic epithelial cells. [48]. In case of *PDE4B*, the administration of a PDE inhibitor in glaucoma rat models induced attenuation of neuroinflammation and enhanced viability in retinal ganglion cells [49]. Experiments in mouse models with a superoxide dismutase *SOD1* knockout suggested that its deficiency provokes loss of retinal ganglion cells in normal-tension glaucoma (NTG) [50]. Further analysis indicated that SOD1 serum levels were significantly lower in NTG patients when compared with healthy controls.

The 44 genes with at least two interactions with the trace elements in Table 1 are associated with L1 signal transduction, chromatin modifying enzymes, DDX58/IFIH1-mediated activation of interferon-alpha/beta, and MET pathway negative regulation. Mutations in the IFIH1 and DDX58 genes are linked to the Singleton-Merten syndrome, which can manifest as glaucoma [55]. Also, imbalance in cytokine production by T helper cells 1 and 2 is known to be involved in neural damage and glaucoma [56].

## Limitations

Our web-based application provides a user-friendly graphical interface to search for trace element-binding proteins, as well as their associated pathway and biology term in glaucoma disease. New features and additional customisation of the visualisations would enhance Glaucoma-TrEl. We will add the variation with the clinical validation report and enrichment analysis in the future.

## Data Availability

Project Name: Glaucoma TrEl. Project home page: www.jveralab.net/glaucoma-trel/. Operating Systems: Platform-independent. Programming language: R. Other requirements: internet connection, internet browser. License: CC-BY-4.0. Any restrictions to use by non-academics: No.

## List of abbreviations

APP: Amyloid precursor protein.
B2M: Beta-2-Microglobulin.
BP: biological process.
Cd: cadmium.
Co: cobalt.
Cu: copper.
EBI-EMBL: European Bioinformatics Institute- European Molecular Biology Laboratory.
FCG: feline congenital glaucoma.
Fe: iron.
GDNF: glia cell-derived neurotrophic factor.
GO: gene ontologies.
HDAC8: Histone Deacetylase 8.
IOP: intraocular pressure.
MET pathway: Mesenchymal-Epithelial Transition pathway.
MHC-I: Major histocompatibility complex class I.
MINAS: Metal Ions in Nucleic AcidS.
MMDB: Molecular Modeling Database.
Mn: manganese.
NTG: Normal-tension glaucoma.
ON: Optic nerve.
PDB: Protein Data Bank.
PDBe: Protein Data Bank in Europe.
PDE4B: Phosphodiesterase 4B.
PMID: Public/Publisher MEDLINE.
POLH: DNA Polymerase Eta.
RCSB: Research Collaboratory for Structural Bioinformatics.
RET: rearranged during transfection.
SAR: Surface abnormality ratio.
SNRPA: Small Nuclear Ribonucleoprotein Polypeptide A.
SOD1: Superoxide Dismutase 1.
STRING: Search Tool for the Retrieval of Interacting Genes/Proteins.
TM: trabecular meshwork.
TPM: transcript per million.
UBC: gene ubiquitin C.
UniProt: Universal Protein Resource.
Zn: zink.
